# Performance Degradation of Amine-Infused Fiber Sorbents for Direct Air Capture: Mechanisms and Solutions

**DOI:** 10.1021/acs.iecr.5c00462

**Published:** 2025-06-17

**Authors:** Yuxiang Wang, João Marreiros, Joshua A. Thompson, Todd J. Toops, Zachary S. Campbell, Michelle K. Kidder, Christopher J. Janke, Jia Qing Leow, David S. Sholl, Ryan P. Lively

**Affiliations:** † School of Chemical & Biomolecular Engineering, 1372 Georgia Institute of Technology, 311 Ferst Dr., Atlanta, Georgia 30332, United States; ‡ 6146 Oak Ridge National Laboratory, Oak Ridge, Tennessee 37830, United States

## Abstract

Sorbent stability poses significant impacts on long-term performance of direct air capture (DAC) of CO_2_ and levelized cost of capture (LCOC). We report the DAC performance degradation of amine-infused fiber sorbents based on poly­(ethylenimine) (PEI), mesoporous SiO_2_, and cellulose acetate (CA) over CO_2_ cyclic sorption cycles in a nonoxidative environment. Infrared and nuclear magnetic resonance spectra indicate that the aminolysis reactions between CA ester moieties and PEI amine sites lead to the formation of acetamides and hence lower CO_2_ affinities of the sorbents. This stability issue can be remedied by hydrolysis treatment of the CA fiber sorbents before PEI impregnation or replacing CA with poly­(ether sulfone). This study underscores the importance of selecting proper support or additive materials of DAC contactors that are compatible with active species of CO_2_ capture.

## Graphical abstract

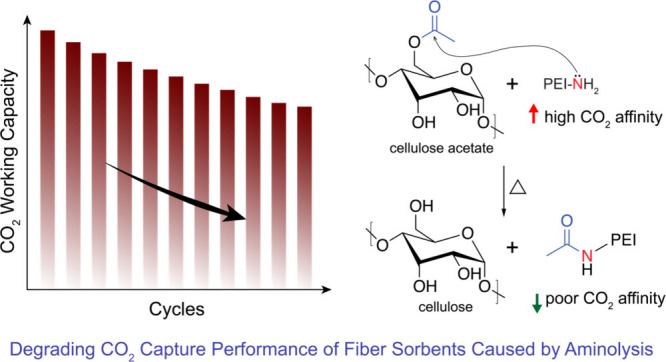

## 1 Introduction

Direct air capture (DAC) is a key negative emission technology and focuses on the removal of CO_2_ from the atmosphere.^1^ One significant design parameter for solid sorbent DAC processes is the sorption contactor where sorbent materials are supported and CO_2_ adsorption/desorption occurs. Ideally, the DAC contactors should be affordable at large production scales to minimize the capital costs. These contactors should also contain as much sorbent as possible to maximize the CO_2_ uptake capacities while achieving fast mass and heat transfer kinetics so that the CO_2_ productivity of the process (i.e., moles of CO_2_ captured per unit volume of the DAC system per unit time) can be optimized. In addition, the design of sorption contactors has significant influences on pressure drop during CO_2_ adsorption, which further affects the operational cost of DAC processes. Despite the unfavorable high pressure drop and energy penalty associated with high air velocities,^2^ recent theoretical and experimental studies suggest that increasing air velocity through DAC sorption contactors helps to increase the amount of CO_2_ fed into the system and consequently achieve higher productivity despite the shorter residence time of CO_2_ in the system.^3,4^

Contactors in forms of monoliths,^5–7^ laminates,^8,9^ and fibers have been developed for DAC applications.^10–12^ Additional substrate or binder materials are required to integrate or shape DAC adsorbents into the contactors. These materials are typically ceramic materials such as Al_2_O_3_ and SiO_2_,^13–17^ carbon materials such as activated carbons,^18^ metal oxides,^19,20^ polymeric materials,^21–23^ and microporous materials such as metal–organic frameworks (MOFs).^24,25^ As these materials are in the vicinity of the active components (i.e., amines in most scenarios) for DAC, physical and chemical compatibility between material components is crucial for achieving robust CO_2_ capture performance over long-term operation. Poor physical and chemical compatibility under DAC operating conditions might lead to deactivation of CO_2_ sorption sites and deterioration in CO_2_ capture performance of the contactors over extended applications, which will require more frequent sorbent replacement and lead to higher levelized cost of carbon capture (LCOC) according to prior techno-economic analyses.^26^ Despite the well-established research interests in the stability and degradation mechanisms of amines in oxidative environments,^27–30^ a shortage of publications has evaluated the stability of amines in the context of sorption contactors.

This work investigates the deterioration of CO_2_ capture performance of amine-infused cellulose acetate (CA)/SiO_2_ fiber sorbents over multiple CO_2_ adsorption/desorption cycles. CA has been adopted to spin fiber sorbents containing porous supports such as silica and metal–organic frameworks. Previous works show that amine-infused CA fiber sorbents possess excellent CO_2_ uptake capacities and kinetics at both ambient and subambient conditions,^10,11^ yet the long-term DAC performance of these fiber sorbents is not fully understood. We report here a degradation mechanism for amine-infused CA fiber sorbents based on the aminolysis reactions between amines and CA ester moieties. Identification of this mechanism allows us to propose two approaches to significantly improve the performance robustness of amine-infused fiber sorbents for CO_2_ capture.

## 2 Results and Discussion

Fiber adsorbents are a promising group of adsorption contactors that strike a good balance between high sorbent loading, fast mass transfer kinetics, low pressure drop, and low manufacturing cost.^12,31^ In these fiber sorbents, micro- or mesoporous adsorbent particles are homogeneously distributed in macroporous polymer matrices to form “sieve in cage” textures. Branched poly­(ethylenimine) (PEI, *M*_w_ = 800 g mol^–1^) has been infused in various fiber sorbents based on different polymeric binders such as cellulose acetate (CA), Torlon, and PIM-1, imparting the fiber adsorbents with excellent CO_2_ capture capacity.^10,32–34^ In this study, fiber sorbents based on CA and amorphous mesoporous SiO_2_ (denoted as CA/SiO_2_) were spun by a dry-jet-wet-quench spinning method reported previously (Table S1).^10,35^ The SiO_2_ particles are evenly distributed in the macroporous CA matrix as shown by the scanning electronic microscopy (SEM) (Figure S1), and the porous surface morphology of the CA/SiO_2_ fibers was realized by coextruding a sheath solution of NMP/H_2_O mixture along with the main polymer dope during the spinning process. SiO_2_ composition in the fibers is about 48 wt % based on thermogravimetric analysis (TGA) (Figure S2). The Brunauer–Emmett–Teller (BET) surface area and pore volume (measured at 0.95 P P_0_^–1^) of the SiO_2_ are 259 m^2^ g^–1^ and 1.2 cm^3^ g^–1^, respectively. These values decrease to 130 m^2^ g^–1^ and 0.62 cm^3^ g^–1^ for the BET surface area and pore volume (estimated at 0.95 P P_0_^–1^), respectively, in the CA/SiO_2_ fibers due to the addition of CA. (Figure S3a). PEI was infused into the fibers after the fiber spinning process via the procedures reported in prior works,^4,36^ and the amine-loaded fiber sorbents are denoted as CA/SiO_2_/PEI. Compared to CA/SiO_2_, the porosity of CA/SiO_2_/PEI decreases significantly as PEI occupies the pore volume of SiO_2_ (Figure S3b).^4^ These fiber sorbents were packed into adsorption columns for breakthrough experiments using 400 ppm CO_2_ at 40% RH and 35 °C. Interestingly, a significant deterioration of CO_2_ uptake capacities in the fibers (27% capacity loss) was observed over 15 cycles of breakthrough experiments even though the fibers were not exposed to air in either adsorption or desorption steps (Figure S4). Repeating the breakthrough experiments using different batches of CA/SiO_2_/PEI fibers and different breakthrough setups confirmed the trend of performance degradation (Figure S4).

To identify the mechanism accounting for the degraded CO_2_ uptake performance, a testing protocol based on a TGA was developed to record CO_2_ uptake capacities at pseudoequilibrium of a series of controlled samples over automated adsorption–desorption cycles (Figure S5a). Detailed testing protocol is available in the Supporting Information. In brief, the activated sample was purged by 400 ppm CO_2_ with 2.0 – 2.1% H_2_O (35–38% RH) and N_2_ balance at 35 °C for adsorption and 90 °C for desorption alternately. The CO_2_ and H_2_O compositions at the outlet of the furnace were recorded by an infrared gas composition analyzer. The CO_2_ desorption concentration profile (Figure S5b) of each desorption step was further processed to derive the desorbed amount of CO_2_, which can be considered as the CO_2_ working capacities of the adsorbents between the adsorption and desorption conditions.

As shown in Figure 1a, CA/SiO_2_/PEI with a PEI loading of 1.0 g_PEI_ g_SiO2_^–1^ (determined by TGA, Figure S6) lost about 40% CO_2_ working capacity over 20 cycles from 1.1 to 0.58 mmol g^–1^, the trend of which is consistent with the degraded performance observed in breakthrough experiments. When the PEI loading is 0.46 g_PEI_ g_SiO2_^–1^ in the fibers (Figure S6), the performance decline was even more pronounced, up to 60% over 20 cycles. These losses in CO_2_ uptake are comparable or even more severe than the cases where the amine sorbents are exposed to O_2_.^28,37^ Because prior studies have revealed that the porosities of fiber sorbents decrease with increasing PEI loadings,^4^ these results also suggest that the significant drop of CO_2_ uptake capacities of CA/SiO_2_/PEI is independent of PEI loadings and internal porosities. Although no O_2_ was introduced to the furnace during the experiments, air might leak into the furnace and cause amine degradation in the presence of trace O_2_. However, the changes of sample masses after the CO_2_ desorption steps were minimal (Figure S7), suggesting that the extent of amine mass loss was negligible. This interpretation was further corroborated by cyclic sorption experiments of SiO_2_/PEI that showed robust CO_2_ uptake performance (Figure S8). Interestingly, the CO_2_ working capacities in CA/SiO_2_/PEI became significantly lower with slightly better cycle stability after the fibers were preheated at 110 °C under ultrahigh vacuum (∼ 20 mTorr) (Figure 1b). Taken together these results indicate a degradation pathway without the involvement of O_2_ accounts for the deterioration of CO_2_ working capacities of these fiber sorbents.

**Figure 1:**
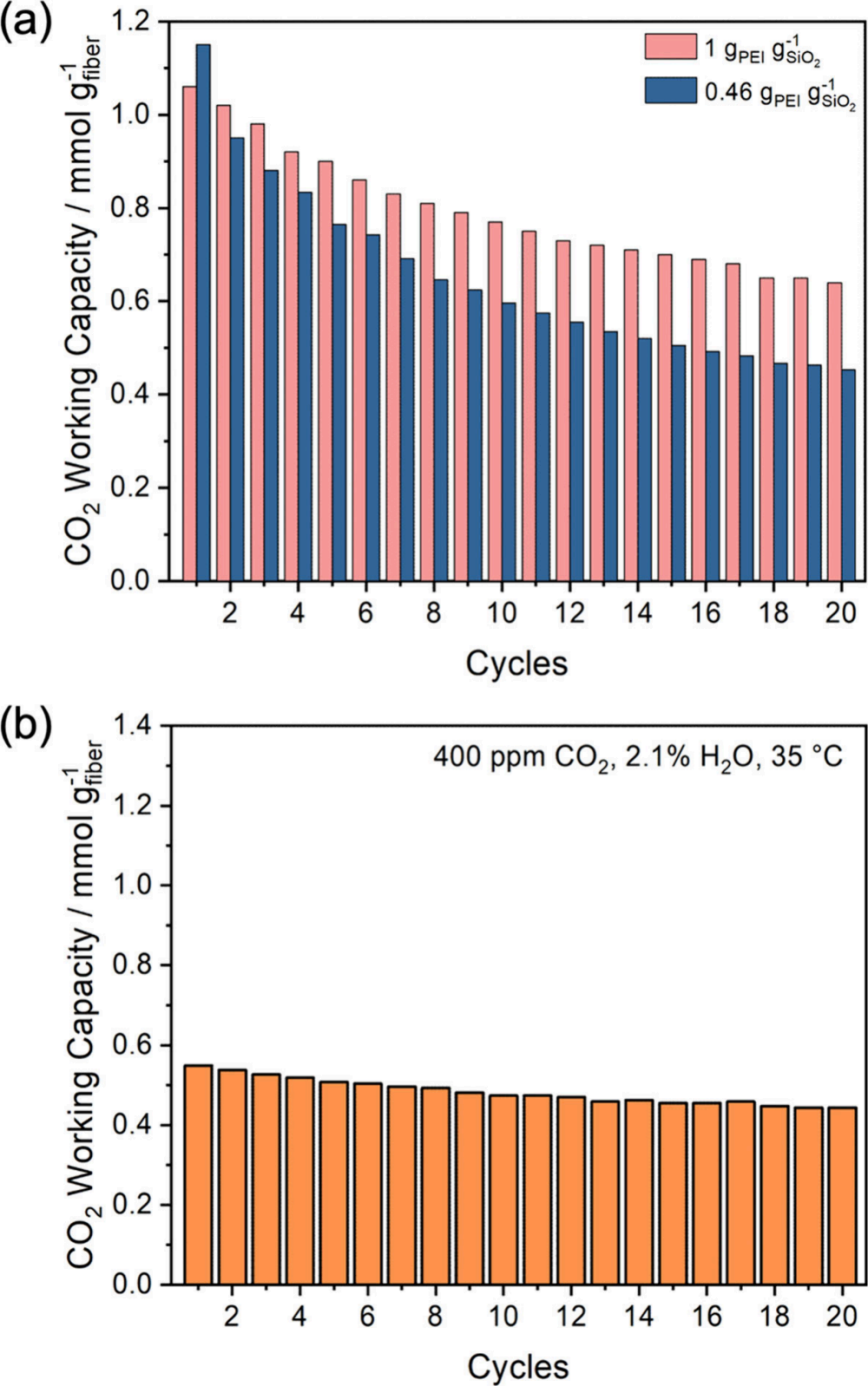
(a) CO_2_ working capacities of CA/SiO_2_/PEI with different PEI loadings. (b) CO_2_ working capacities of CA/SiO_2_/PEI (1.0 g_PEI_ g_SiO2_^–1^) after activation in vacuum at 110 °C. In the cyclic CO_2_ adsorption–desorption experiment, the adsorption condition is 400 ppm CO_2_ and 2.0% H_2_O in N_2_ at 35 °C, while the desorption condition is 400 ppm CO_2_ and 2.0% H_2_O in N_2_ at 90 °C. Data in the figures are available in Table S2.

A series of characterizations based on attenuated total reflection Fourier transform infrared (ATR-FTIR) spectroscopy and nuclear magnetic resonance (NMR) were conducted to better understand the degradation mechanism. Figure 2a shows the ATR-FTIR spectra of CA/SiO_2_/PEI and other control samples before and after the cyclic CO_2_ adsorption/desorption experiments. CA and CA/SiO_2_ fibers share the same characteristic IR bands highlighted by the gray areas, where the characteristic bands corresponding to the stretching vibration of C=O from the CA ester moieties are located at 1746 cm^–1^. New bands highlighted by blue areas indicate the presence of PEI in the fiber sorbents after PEI infusion. A shoulder at 1644 cm^–1^ highlighted in the yellow area appears along with other PEI characteristic peaks at 1559 (N–H bending) and 1467 cm^–1^ (C–H bending) in the spectrum of the fresh CA/SiO_2_/PEI sample.^38^ After the sorption cycle experiments, the intensity of the C=O IR band at 1746 cm^–1^ decreases dramatically for the CA/SiO_2_/PEI, indicating a reduced concentration of ester C=O groups in the sample. A similar decline in intensity for the peak at 1746 cm^–1^ and intensity increment for the peak at 1644 cm^–1^ also appear in the spectrum of CA/SiO_2_/PEI after 110 °C treatment in vacuum. In comparison, there are no significant differences between the ATR-FTIR spectra of SiO_2_/PEI composites before and after the cyclic sorption experiments (Figure S9). The changes in ATR-FTIR spectra of CA/SiO_2_/PEI after CO_2_ sorption cycles suggest that the loss in CO_2_ working capacities is related to the consumption of CA ester groups. As the C=O IR peak at 1746 cm^–1^ declined with the concomitant increase in intensity of the IR band at 1644 cm^–1^, it is likely that the ester groups in CA were converted to other functional groups with C=O moieties.

**Figure 2:**
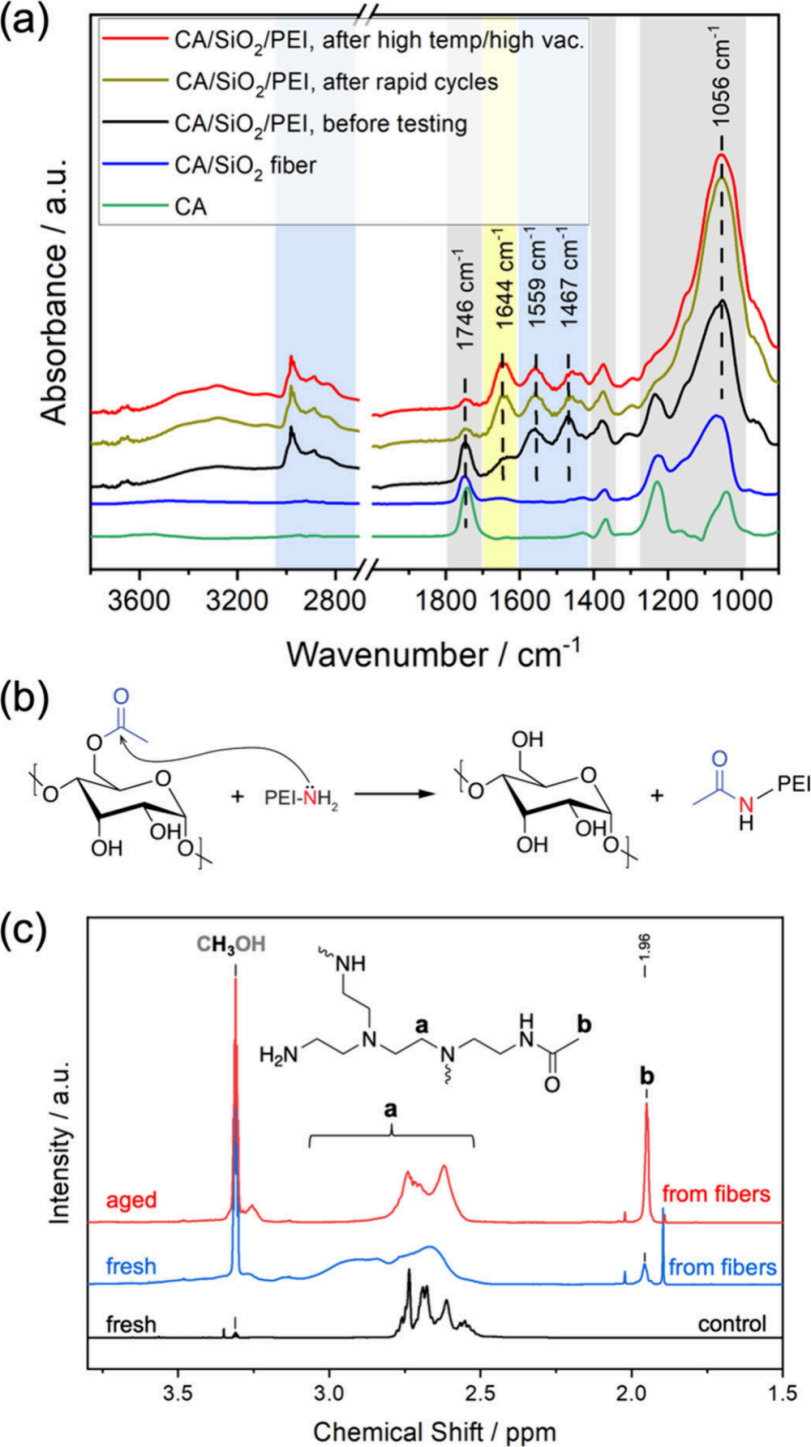
(a) ATR-FTIR spectra of CA/SiO_2_, fresh CA/SiO_2_/PEI, CA/SiO_2_/PEI after cyclic CO_2_ adsorption/desorption cycles, and aged CA/SiO_2_/PEI after overnight soaking at 110 °C in vacuum. PEI loading in these CA/SiO_2_/PEI fibers was 1.0 g_PEI_ g_SiO2_^–1^. The characteristic ATR-FTIR bands of PEI and CA are highlighted in blue and gray areas, respectively. (b) The proposed reaction mechanism between PEI and CA. (c) ^1^H NMR spectra of MeOH-d_4_ extraction of fresh CA/SiO_2_/PEI and CA/SiO_2_/PEI fibers after CO_2_ adsorption/desorption cycles. The ^1^H NMR spectrum of fresh PEI dissolved in MeOH-d4 is shown for comparison.

Considering the abundance of primary amines in the fiber sorbents and the greater nucleophilicity of primary amines compared to hydroxyl groups, we hypothesize that an aminolysis reaction of the CA ester groups accounts for the performance degradation of CA/SiO_2_/PEI (Figure 2b). When the fiber sorbents are heated during desorption, the PEI chains originally in the pores of SiO_2_ likely gain mobility to potentially reach the CA network, making it possible for the primary amines to attack the ester moieties of CA. As a result, the CA ester C=O IR band at 1746 cm^–1^ becomes weaker, while the amide C=O band at 1644 cm^–1^ becomes stronger. Primary amines, which have a high CO_2_ affinity, are converted to acetamide, whose N basicity and CO_2_ affinity are lower due to conjugation.

Further ATR-FTIR and NMR spectra were collected to test our hypothesized mechanism. If ester groups were transferred from CA to PEI, the IR peak corresponding to the amide carbonyl groups should disappear in the IR spectrum of the fiber sorbents after the guest PEI polymers are removed by extraction. Indeed, after washing by deuterated methanol (MeOH-d_4_), a good solvent for PEI and PEI derivatives but poor solvent for CA, the IR peak attributed to the new carbonyl species at 1644 cm^–1^ disappears along with the PEI bands in the range of 1600–1400 cm^–1^ in the ATR-FTIR spectrum of CA/SiO_2_/PEI after CO_2_ sorption/desorption cycles (Figure S10). In addition, the IR peak of CA carbonyl groups does not recover after MeOH washing, confirming the irreversible transformation of the CA binder in the fiber sorbents. The ^1^H NMR spectrum of MeOH-d_4_ extraction of the aged fiber sorbents reveals the characteristic methyl protons from the acetyl moieties apart from the methylene protons of PEI (Figure 2c). In comparison, the methyl proton peak in the ^1^H NMR spectrum of MeOH-d_4_ extraction of fresh CA/SiO_2_/PEI is marginal compared to the high peaks of PEI methylene protons. These results collectively indicate that the migration of acetyl groups from CA to PEI during the sorption cycles accounts for the dwindling CO_2_ uptake capacities in CA/SiO_2_/PEI. The reaction between CA and PEI could even take place during the PEI infusion process at room temperature, as suggested by the shoulder IR peak at 1644 cm^–1^ highlighted by the yellow area in the ATR-FTIR spectrum (Figure 2a) and the small methyl proton peak in ^1^H NMR spectrum of the MeOH-d_4_ extraction of fresh CA/SiO_2_/PEI (Figure 2c). The reaction rate presumably becomes much faster at the elevated temperatures used for sorbent regeneration, and the accumulated time at the activation temperatures leads to the high extent of ester–amide conversion and the pronounced deterioration of CO_2_ uptake capacities.

In addition to ^1^H NMR analysis of MeOH-d_4_ extraction of the fibers, complementary solid-state ^13^C NMR analysis of fresh CA/SiO_2_/PEI was performed. Figure S11a verified the presence of sharp characteristic signals attributed to CA, namely peaks at 171.5 ppm assigned to C=O, and peaks at 20.6 and 22.5 ppm assigned to −CH_3_, from acetate functional groups in line with prior reports.^39,40^ These peaks are also accompanied by fingerprint signals of PEI, comprised in the range 37–58 ppm. After overnight heating at 90 °C, the spectrum of the same sample revealed a number of changes to CA and PEI identifying signals. The previously recorded C=O signal shows a small loss of intensity accompanied by considerable broadening (chemical shift anisotropy) and an upfield shift to 107.7 ppm, which is in line with modification of the chemical environment of the carbonyl groups as suggested by our aminolysis hypothesis. Similar changes are also observed for −CH_3_ signals whose shifting however downfield to 21.7 and 23.3 ppm, respectively, suggesting an increase in electronic shielding surrounding these functional groups. Meanwhile, PEI fingerprint signals record substantial broadening accompanied by downfield shift in the range of 38.4 to 58.5 ppm, which again are consistent with increased electronic shielding effects, an effect not observed for control samples of SiO_2_/PEI subjected to identical processing and characterizations (Figure S11b).

Since the aminolysis reaction between PEI and CA results in the decline in CO_2_ affinity of the fiber sorbents, we hypothesized that the removal of CA ester groups prior to PEI infusion can help preserve the active primary amine sites in the fiber sorbents. Therefore, CA/SiO_2_ fibers were hydrolyzed in 0.1 M KOH aqueous solution before PEI infusion, and the PEI-loaded fiber sorbents after hydrolysis (2.1 g_PEI_ g_SiO2_^–1^, Figure S12a), denoted as CA/SiO_2_–KOH/PEI, were subjected to the CO_2_ cyclic sorption experiments. ATR-FTIR spectra confirm that most CA ester groups were removed by the hydrolysis step according to the significantly attenuated ester carbonyl peak shown in Figure 3a. Moreover, minimal primary amines were converted to amides after cyclic experiments according to the IR and NMR analysis (Figure S12b). In terms of CO_2_ capture performance, the initial CO_2_ working capacity of CA/SiO_2_–KOH/PEI was 1.64 mmol g^–1^ as shown in Figure 3b, which is more than 40% higher than the counterparts of CA/SiO_2_/PEI regardless of the PEI compositions (Figure 1a). CA/SiO_2_–KOH/PEI also exhibited more robust CO_2_ working capacities over sorption cycles, maintaining more than 85% of the peak capacity versus 68% for CA/SiO_2_/PEI (1.0 g_PEI_ g_SiO2_^–1^) at the end of the TGA experiments. The substantially improved stability of the fiber sorbents suggests the effectiveness of this approach, although the residual ester groups in the fiber sorbents due to the difficulty of achieving 100% hydrolysis might still result in the observed loss in CO_2_ working capacities.

**Figure 3:**
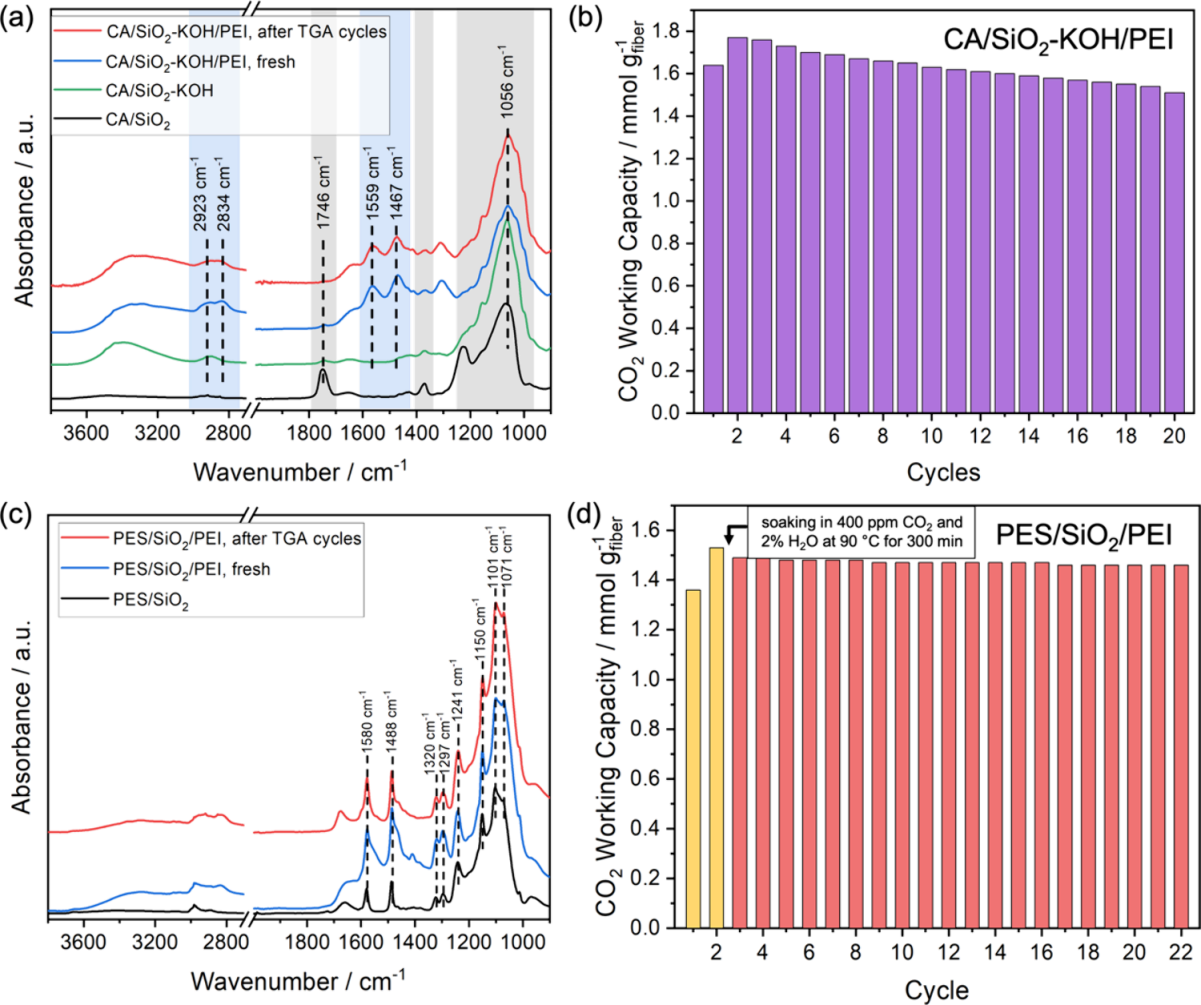
(a) ATR-FTIR spectrum of CA/SiO_2_, CA/SiO_2_–KOH, fresh CA/SiO_2_–KOH/PEI, and CA/SiO_2_–KOH/PEI after CO_2_ adsorption–desorption cycles. The characteristic ATR-FTIR bands of PEI and CA are highlighted in blue and gray areas, respectively. (b) CO_2_ working capacities of CA/SiO_2_–KOH/PEI fiber adsorbents during the cyclic CO_2_ adsorption–desorption experiment. (c) ATR-FTIR spectrum of PES/SiO_2_, fresh PES/SiO_2_/PEI, and PES/SiO_2_ /PEI after CO_2_ adsorption–desorption cycles. (d) CO_2_ working capacities of PES/SiO_2_ /PEI fiber adsorbents during the cyclic CO_2_ adsorption–desorption experiment. In the cyclic CO_2_ adsorption–desorption experiment, the adsorption condition is 400 ppm CO_2_ and 2.0% H_2_O in N_2_ at 35 °C, while the desorption condition is 400 ppm CO_2_ and 2.0% H_2_O in N_2_ at 90 °C. The yellow and red columns stand for the CO_2_ working capacities before and after the aging of the fibers in 400 ppm of CO_2_ and 2.0% H_2_O at 90 °C. Data in the figures are available in Table S3.

In addition to the above chemical approach to modify the fiber sorbents before PEI infusion, replacement of CA with other polymers such as poly­(ether sulfone) (PES), an affordable polymer widely used to fabricate membranes and filters, could also address the stability issue of DAC fiber sorbents. The inert polymer backbones of PES with respect to amines should help enhance the stability of the fiber sorbents relative to CA fibers and achieve robust CO_2_ working capacities during CO_2_ sorption cycles. PEI was infused into PES/SiO_2_ fibers like other fibers based on CA, and the PEI loading was 1.1 g_PEI_ g_SiO2_^–1^ (Figure S13a). Figure 3c shows the ATR-FTIR spectra of PES fiber sorbents. The characteristic IR bands for PEI overlap with the counterparts of PES.^41^ No significant changes in IR spectra were observed for the PES/SiO_2_/PEI sorbents after 20 CO_2_ sorption–desorption cycles. The robustness of PES/SiO_2_/PEI fibers were further tested after soaking the fibers at 90 °C for 300 min (equivalent to 20 activation steps in the CO_2_ cyclic sorption experiment) in a humid CO_2_/H_2_O environment. Despite the extended activation period at high temperatures, the fibers showed a CO_2_ working capacity of 1.46 mmol g^–1^ after 20 sorption cycles, which is 97% of the peak value before the extended treatment at 90 °C (Figure 3d). The ^1^H NMR spectrum of MeOH-d_4_ extraction the PES/SiO_2_/PEI fibers after the TGA experiments only contains the peaks of methylene protons (Figure S13b), suggesting that the PEI in the fibers remain stable over the rapid CO_2_ sorption cycles.

## 3 Conclusions

This work systematically investigated the degradation mechanism of CA/SiO_2_/PEI fiber sorbents in an O_2_-free environment. ATR-FTIR and NMR analyses collectively indicate that the aminolysis reactions between the primary amines of PEI and the ester groups of CA lead to the formation of amide groups and reduced CO_2_ uptake capacities in the fiber sorbents. Hydrolysis of CA/SiO_2_ fibers before PEI infusion can minimize the consumption of primary amines due to aminolysis and preserve 85% of the CO_2_ working capacity after 20 adsorption–desorption cycles. An alternative approach to mitigate this degradation mechanism is to replace CA with PES as the latter polymer binder eliminates the possibility of aminolysis reaction and lead to 97% retention of CO_2_ working capacity under the same testing conditions. This work highlights the importance of evaluating the stability of active ingredients (PEI in this study) for CO_2_ capture in the presence of binders or contactors in addition to the evaluation of their resistance against oxidation under DAC-relevant conditions. It is critical to design DAC contactors judiciously to ensure good compatibility between the CO_2_ sorption sites and other components in the contactors to achieve efficient and robust long-term performance for overall DAC systems. A limitation of this work is that the effects of different polymeric binders on the oxidation stability of PEI are not fully understood. In addition, polymer binders or other additives in the sorption contactor will impact the total water uptake of the DAC system during the CO_2_ adsorption step, which will require the adoption of different water management strategies of the system and hence result in different energy intensities and costs of the processes. Future endeavors are necessary for the comprehensive evaluation of the long-term performance of DAC adsorbents/contactors in practical DAC operation conditions.

## Supplementary Material



## Notes

Material and instrument information, material preparation procedures, data processing procedures of TGA and breakthrough experiments, and additional characterization data (SEM, TGA, ATR-FTIR, NMR) (PDF)
