# Supplementary material for: Performance Degradation of Amine-Infused Fiber Sorbents for Direct Air Capture: Mechanisms and Solutions
Source: Ind Eng Chem Res. 2025 Jun 17;64(26):13512–8. doi: 10.1021/acs.iecr.5c00462 (PMC12232288; doi:10.1021/acs.iecr.5c00462)
Supplement: Supplementary file 1 [file ie5c00462_si_001.pdf]

Supporting information

Performance Degradation of Amine-Infused Fiber Sorbents for Direct Air Capture: Mechanisms and Solutions

Yuxiang Wang,[a] João Marreiros,[a] Joshua A. Thompson,[b] Todd J. Toops,[b] Zachary S. Campbell,[a] Michelle K. Kidder,[b] Christopher J. Janke,[b] Jia Qing Leow,[a] David S. Sholl,[a,b] Ryan P. Lively*,[a]

a. *School of Chemical & Biomolecular Engineering, Georgia Institute of Technology, 311 Ferst Dr., Atlanta, GA 30332, USA*

b. *Oak Ridge National Laboratory, Oak Ridge, TN, USA*

Email: ryan.lively@chbe.gatech.edu

1. Materials and Instruments

1.1. Chemicals. Mesoporous SiO_2 SYLOID® C 803 was purchased from W. R. Grace. Cellulose acetate (CA, M_n ~ 50,000 by GPC, 39.7 wt% acetyl), methanol (ACS reagent), methanol-d_4 (99.8%), and branched poly(ethylenimine) (PEI) (M_w 800 by mass spectrometry) were purchased from Sigma-Aldrich. N-hexane (ACS grade), N-methyl-2-pyrrolidone (NMP, ACS grade), and 50% (w/v) KOH aqueous solution were purchased from VWR BDH Chemicals. Poly(ether sulfone) (PES) Veradel 3000P was provided by Syensqo as a courtesy. All chemicals were used as received. Cylinders of N_2 (99.995%) and 400 ppm CO_2 balanced by N_2 were purchased from Airgas. DI water was obtained from an Elga PureLab Option S7 water purification system.

1.2. Instruments. Scanning electron microscopy (SEM) images were obtained with a Hitachi SU8010. N_2 physisorption experiments at −195.8 °C were measured with a Belsorp MAX (MicrotracBEL, Japan). Prior to isotherm estimation, the samples were degassed under vacuum below 10^{-2} kPa for 12 h at 120 °C. Thermal decomposition experiments to determine SiO_2 loadings in fibers were performed on a TA Instruments TGA Q500 using air as the purging gas. Attenuated total reflection Fourier transform infrared (ATR-FTIR) spectra were collected using a PerkinElmer Spectrum Two. Solution-state proton nuclear magnetic resonance (^{1}H-NMR) spectra were collected with a Bruker AV3 spectrometer operating at 400 MHz. Direct polarization (DP) Solid-state ^{13}C NMR spectra were collected on a Bruker Avance III 400 MHz with a triple resonance MAS probe - samples were spun at 15 kHz in 4 mm rotors, and spectra collected at room temperature.

2. Spinning of Fiber Adsorbents

Polymer fibers containing SiO_2 were spun via the dry-jet, wet quench spinning technique using a custom-built spinning setup. All polymers and silica were dried under 25 in Hg vacuum at 110 °C for 12 hours before being made into the polymer dope. Typical dope compositions are shown in Table S1. During the spinning process, a mixture of NMP/H_2O (75/25 wt%) was co-extruded with the main polymer dope on the sheath side. The typical flow rates for the main polymer dope and the sheath solution are 250 and 40 mL h^{-1}, respectively. More technical details on the spinning procedure can be found in previous literature.[1-3]

Table S1. Compositions of the spinning dopes for CA/SiO_2 and PES/SiO_2 fibers.

Component	Mass / wt%	
	CA/SiO_2	PES/SiO_2
CA	10	
PES		11.6
PVP	4	3.2
SiO_2	12.2	14.2
NMP	65.2	67.8
H_2O	8.6	3.2

3. Infusion of PEI into Fiber Adsorbents

Fibers containing SiO_2 were impregnated with PEI via the following impregnation procedure. About 100 mg of fibers were soaked in a vial containing 5 mL methanol for 30 min. Methanol was then decanted, and the vial was filled with 5 mL PEI/methanol solution (10 – 15 wt% depending on the desirable loading levels of PEI). The vial was placed horizontally during the impregnation. The fibers stayed in the PEI solutions for 4 hours at room temperature (~ 23 °C). Then, the

impregnated fibers were briefly washed by n-hexane for three times before being dried at ambient temperature and pressure overnight.

4. Determination of PEI Loadings in Fibers

PEI loadings in fiber samples were determined using TGA data of PEI-infused fibers and following equation.

$w_{PEI} =$ [illegible] Equation S1

In this equation, m_1 is the first mass fraction plateau in the TGA curve after solvent evaporation at about 110 °C, m_2 is the final mass fraction plateau after heating to 900 °C, and w_{SiO_2} is the mass fraction of SiO_2 in fibers before PEI impregnation.

5. Hydrolysis of CA/SiO_2 Fibers

About 100 mg of CA/SiO_2 fibers were added to a glass vial charged with 5 mL 0.1 M KOH aqueous solution diluted from 50% (w/v) KOH aqueous solution. The mixture was gently shaken overnight, and the fibers were retrieved from the vial and washed by deionized water and MeOH until the supernatant was neutral. The hydrolyzed fibers were subsequently dried at ambient temperature and pressure overnight before further characterizations and amine infusion.

6. Preparation of SiO_2/PEI

PEI impregnation in C803 mesoporous silica was conducted using a procedure reported in the prior work.[4] Briefly, 200 mg of mesoporous silica was first dispersed in 12 mL MeOH. The SiO_2/MeOH suspension was then combined with 3.4 mL of MeOH which contained 200 mg PEI.

The mixture was then stirred for 4 h before removing MeOH by rotary evaporation. The obtained powder was then dried in the fume hood at ambient temperature before further characterizations.

7. Dynamic Breakthrough Experiments

A schematic illustration of the setup for breakthrough experiments is shown in **Scheme S1**. The mass flow controllers (MFCs) in the setup were purchased from Alicat Scientific. The CO_2 and water vapor concentrations at the outlet of adsorption bed were measured by an infrared gas analyzer LI-850-1 produced by LI-COR Environmental, and the relative humidities (RH) in the system were cross-checked by a RH and temperature probe HMP-8 of Vasaila. Humid gas streams were generated by flowing CO_2/N_2 through the customized bubblers that can withstand build-up pressures of the packed bed, and the relative humidities were controlled by adjusting the flow rates of dry and humid streams. The whole setup was placed in a thermalized chamber programmed at specific temperatures to prevent water condensation in the system.

A typical breakthrough experiment is described as follows. PEI-infused fiber sorbents (100 – 200 mg, aspect ratio ~ 100) were packed into a 1/4'' stainless steel column. The bed was activated by 100 sccm dry N_2 at 90 °C overnight until the CO_2 concentrations detected at the outlet of the bed were stable and less than 3 ppm. After activation, the bed was presaturated by a stream of humid N_2 (35% RH) at 35% RH at 35 °C before the breakthrough experiments using a stream of 400 ppm CO_2 balanced by N_2 (100 sccm) with ~ 2% H_2O (35% RH) at 35 °C. The bed was determined to be saturated by the feeding CO_2 when outlet CO_2 concentration was equal to the feeding concentration.

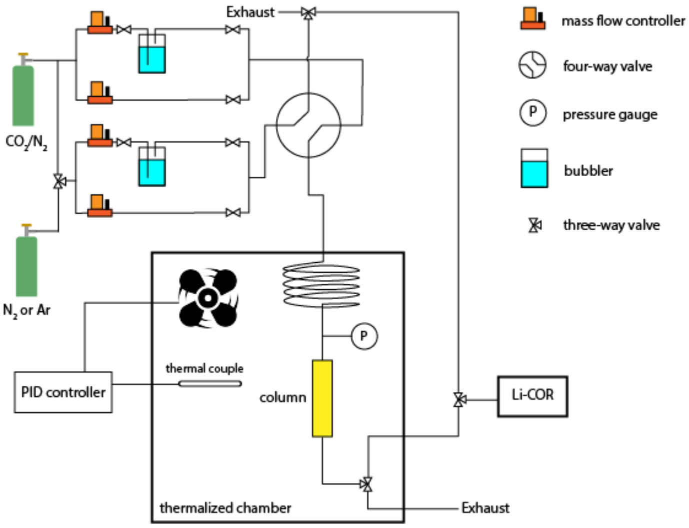

Scheme S1. A scheme of the breakthrough setup used in the study.

The CO_2 uptake capacities, q were calculated based on the breakthrough curves using the following equation.

$$q = \frac{P\dot{V}y_0}{mRT}\int\left(1-\frac{y_t}{y_0}\right)dt \qquad \text{Equation S2}$$

where P is the bed pressure in Pa (pressure drop was negligible), $\dot{V}$ is the volumetric follow rate in $m^3\ s^{-1}$, y_0 is the composition of CO_2 in the feeding gas stream, R is the gas constant, T is temperature in K, m is the sorbent mass in g, and y_t is the CO_2 mol fraction recorded by the gas composition analyzer LI-850-1 at the moment t

8. Automated Rapid CO_2 Cyclic Sorption Experiments

The rapid CO_2 cyclic sorption experiments were performed on a TA Instruments TGA Q500. A LI-COR LI-610 portable dew point generator produced by LI-COR Environmental was used to humidify the CO_2/N_2 streams. An infrared gas analyzer LI-850-1 was connected to the exhaust of the TGA furnace to record CO_2 and H_2O concentrations. About 10 mg of fiber (aspect ratio ~ 10) and powder sample was used in each experiment. The procedure of the rapid CO_2 cyclic sorption experiments is as follows.

Step 1: purge the TGA balance chamber with dry N_2 (200 sccm) for 10 min

Step 2: purge the CO_2 line with 400 ppm CO_2 (300 sccm) for 60 min

Step 3: activate the sample with dry N_2 (200 sccm) at 90 °C for 60 min

Step 4: CO_2 adsorption at 35 °C in 400 ppm CO_2 (dew point 18 °C, 200 sccm) for 60 min

Step 5: CO_2 desorption at 90 °C in 400 ppm CO_2 (dew point 18 °C, 200 sccm) for 15 min

Step 6: repeat step 4 and 5

The CO_2 working capacity, q_w, of each sorption cycle can be determined by the following equation.

$$q_w = \frac{P\dot{V}y_0}{mRT}\int\left(1-\frac{y_t}{y_0}\right)dt \qquad \text{Equation S3}$$

where P is the ambient pressure in Pa, $\dot{V}$ is the volumetric follow rate in m^3 s^{-1}, y_0 is the composition of CO_2 in the feeding gas stream, R is the gas constant, T is temperature in K, m is the sorbent mass in g (the mass after the first activation step), and y_t is the CO_2 mol fraction recorded by the gas composition analyzer LI-850-1 at the moment t Different from gravimetric methods which determine CO_2 uptake capacities based on mass changes of the adsorbent samples,[5] this method avoids the confounding effects of mass changes caused by water adsorption/desorption during CO_2 sorption experiments.

We chose 400 ppm CO_2 flow for the desorption step to simplify the calculation of desorbed CO_2 amount and minimize calculation errors introduced by the large dead volume of the system (TGA plus the dew point generator). If N_2 were used to desorb CO_2, additional blank experiments without any sorbents in the TGA furnace will be necessary for differentiating the effects of gas mixing in the system dead volume from the effects of CO_2 desorption. In comparison, keeping 400 ppm CO_2 mixed gas for the desorption steps minimizes the gas mixing issues and waives the additional blank experiments, thereby allowing us to use Equation S3 to calculate CO_2 working

capacities of the sorbents. In addition, since CO_2 and H_2O are always present in the head space of a DAC bed during practical regeneration steps, we believe that humid CO_2 stream would be an environment that better emulates practical DAC processes compared to pure N_2.

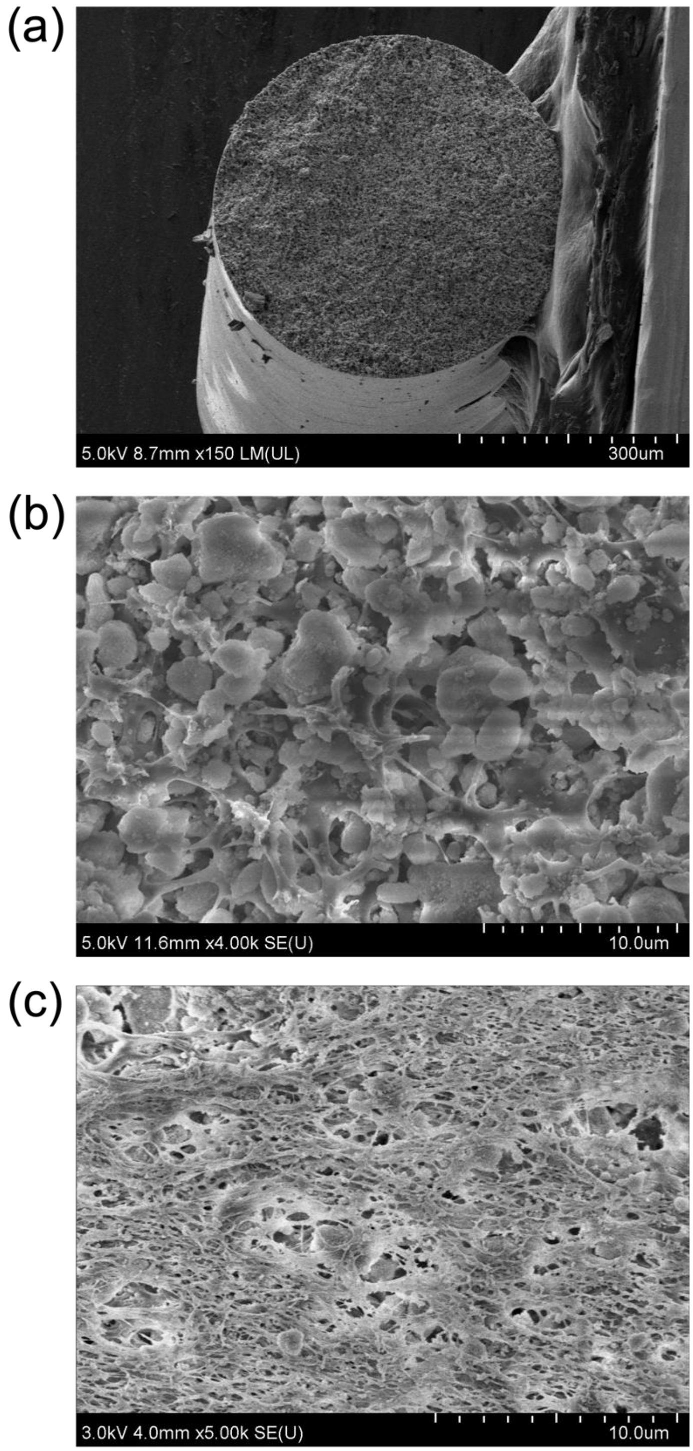

Figure S1. SEM images of CA/SiO_2 fibers. (a-b) shows the cross-section with (a) low and (b) high resolution, while (c) shows the porous surface morphology of the fibers.

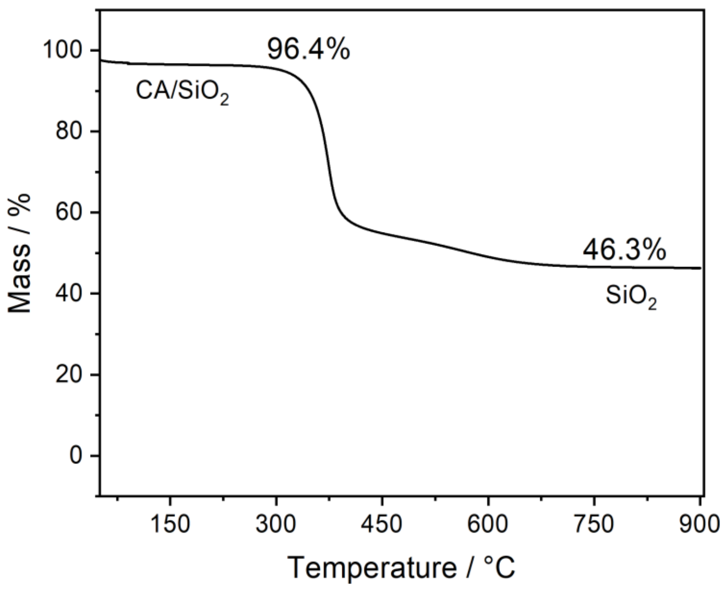

Figure S2. The TGA trace of CA/SiO_2 fibers in air.

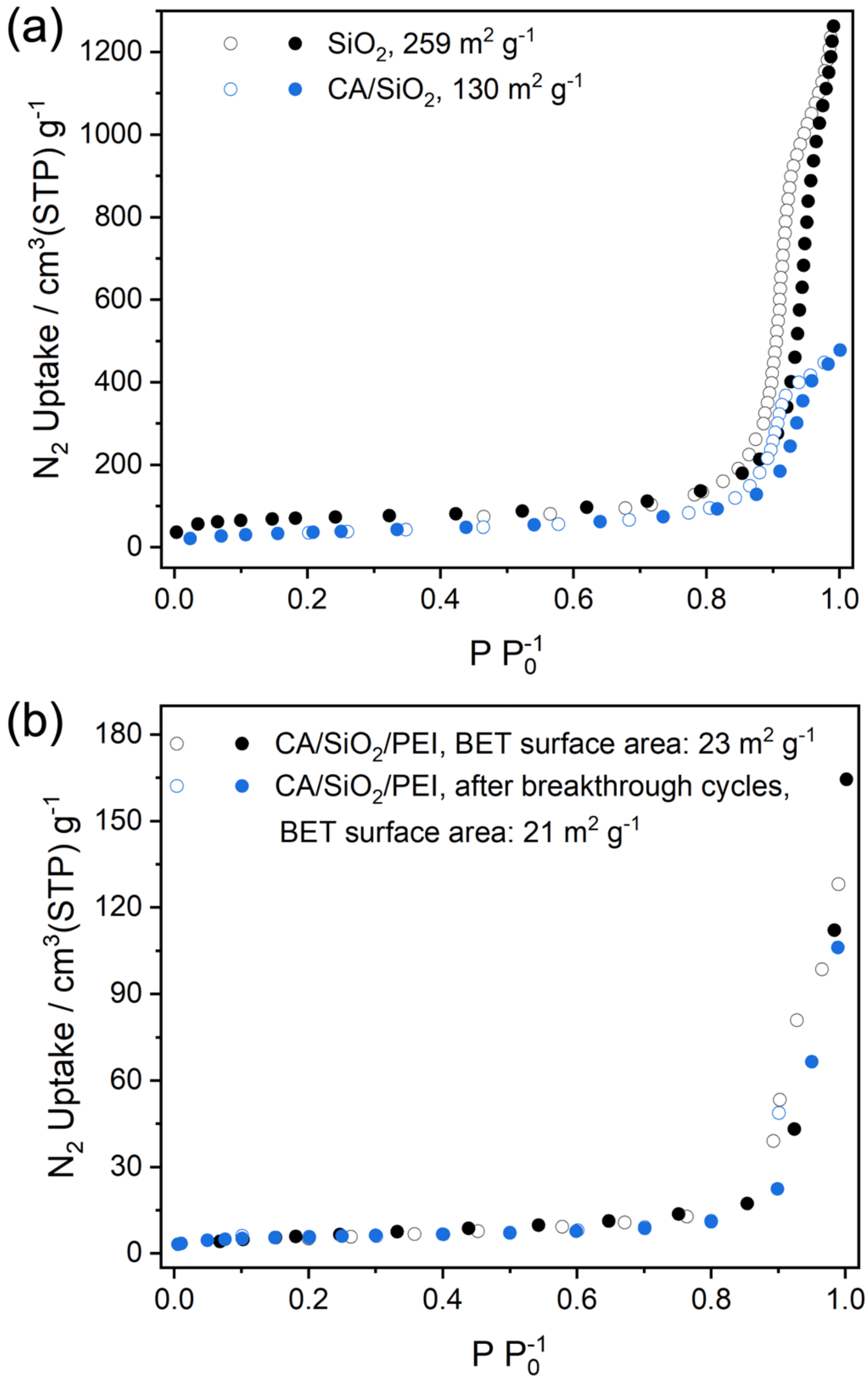

Figure S3. N_2 sorption isotherms at 77 K of (a) SiO_2 and CA/SiO_2 (48 wt% SiO_2 loading), and (b) CA/SiO_2/PEI fibers (0.95 g_{PEI} g_{SiO2}^{-1}) before and after cyclic breakthrough experiments using 400 ppm CO_2/N_2 with 2% H_2O at 35 °C.

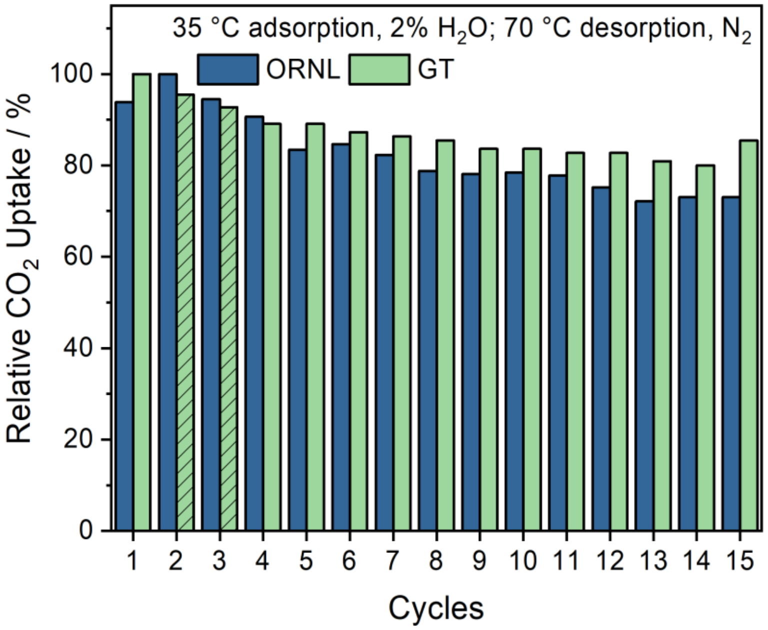

Figure S4. The degradation trends of CO_2 uptake capacities in CA/SiO_2/PEI fibers determined by breakthrough experiments performed at Oak Ridge National Laboratory (ORNL) and Georgia Institute of Technology (GT).

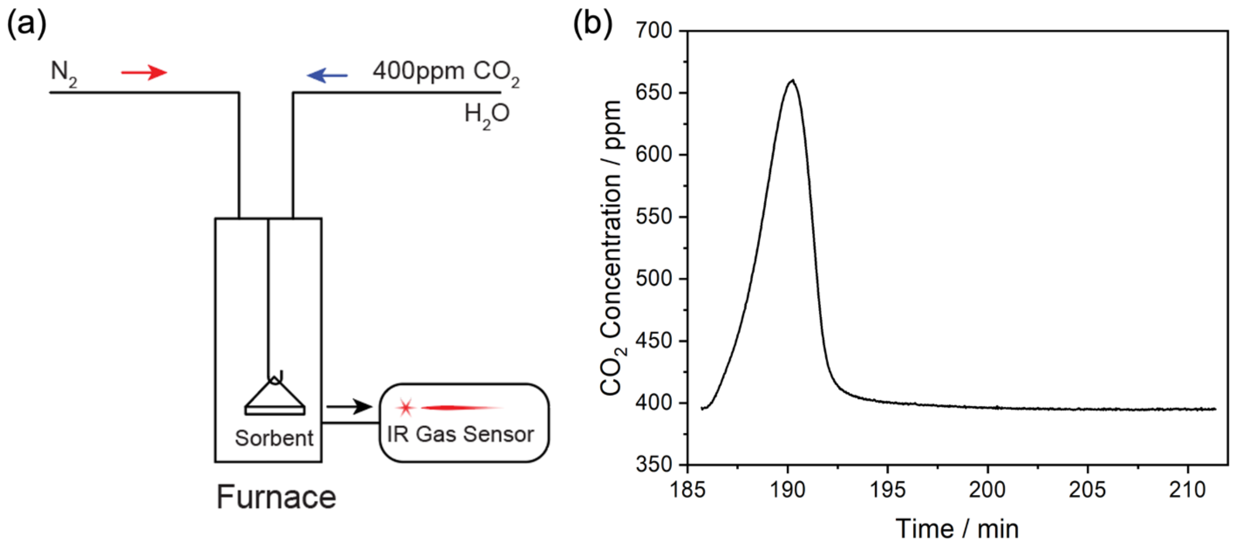

Figure S5. (a) Schematic illustration of the TGA setup for determining CO_2 cyclic sorption performance of adsorbents. (b) CO_2 concentration profile at the exit of TGA furnace in an adsorbent regeneration step.

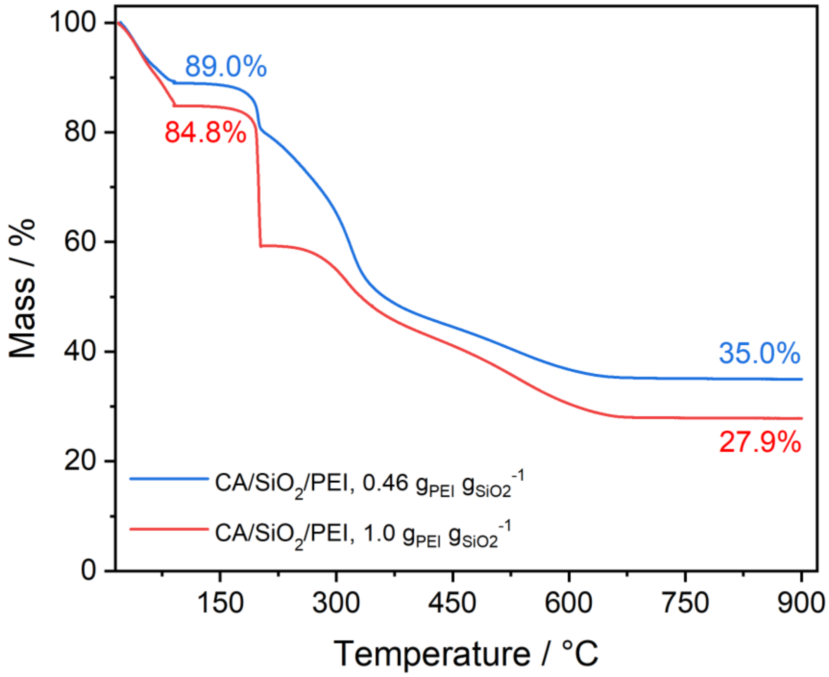

Figure S6. The TGA trace of CA/SiO_2/PEI fibers with different levels of PEI loadings.

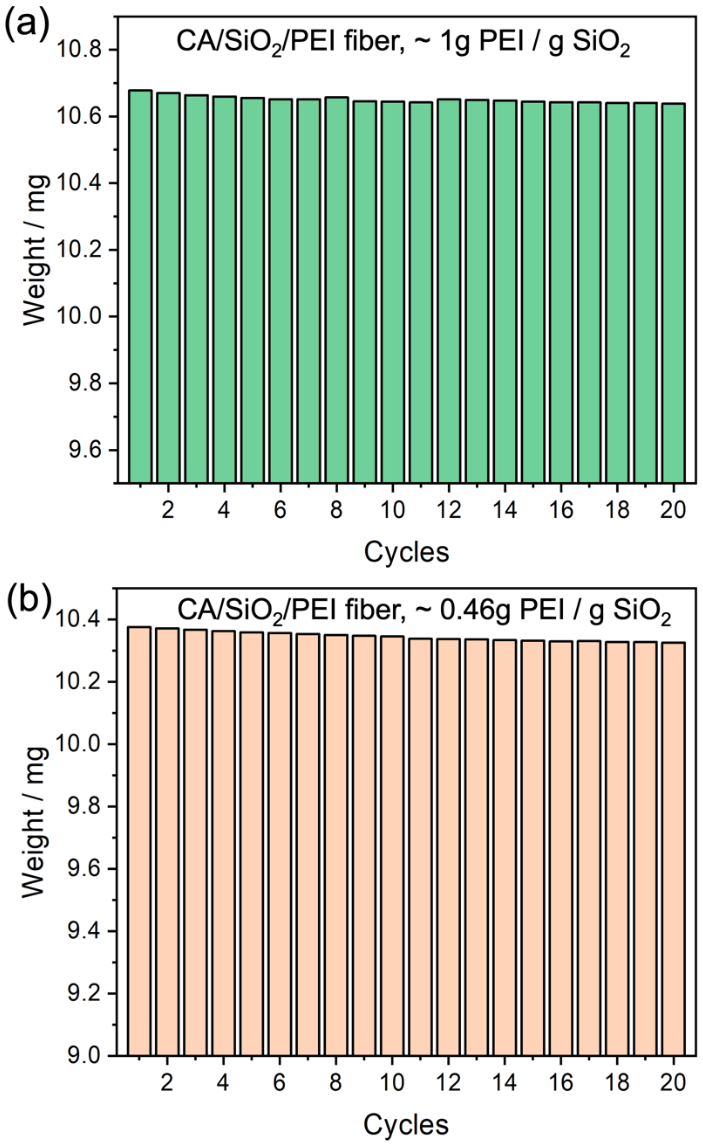

Figure S7. Masses of CA/SiO_2/PEI fiber sorbents with (a) 1.0 g_{PEI} g_{SiO2}^{-1} and (b) 0.46 g_{PEI} g_{SiO2}^{-1} PEI loadings during the cyclic CO_2 adsorption/desorption experiments.

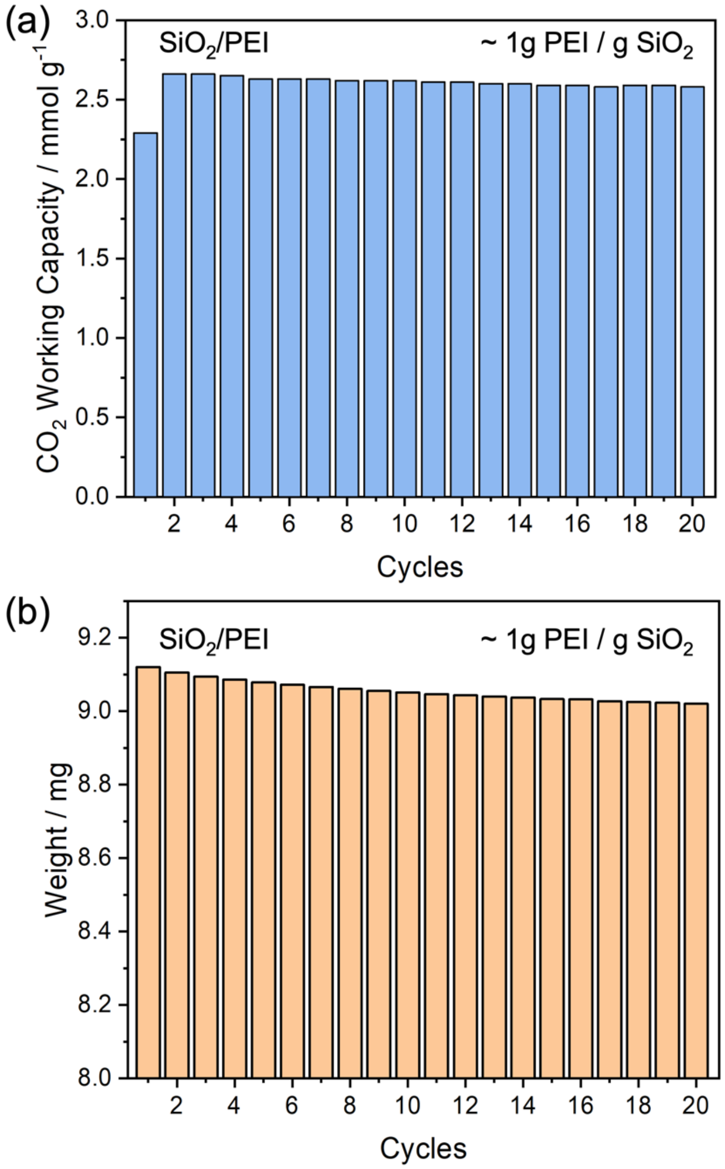

Figure S8. (a) CO_2 working capacities and (b) masses of SiO_2/PEI sorbents during the cyclic CO_2 adsorption/desorption experiments.

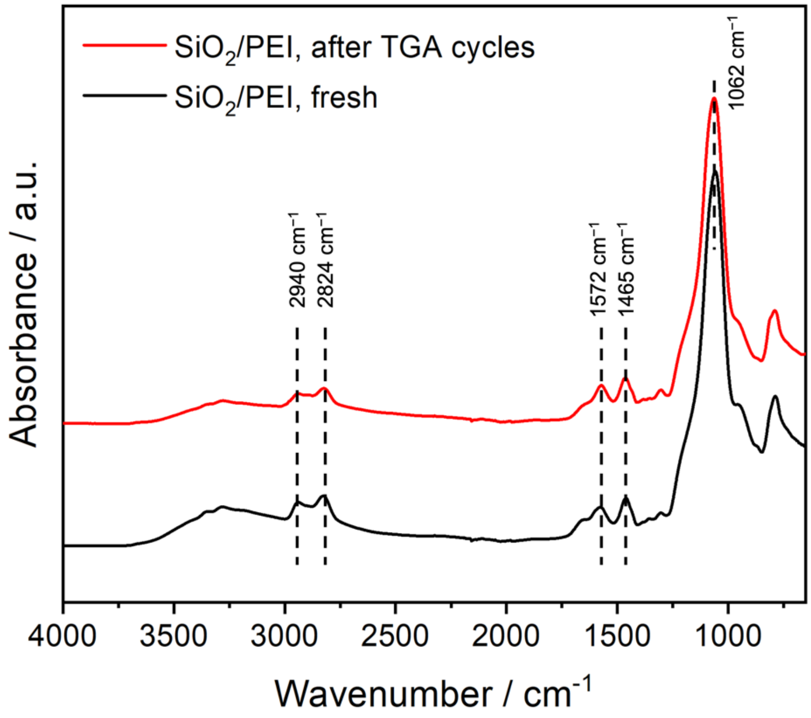

Figure S9. ATR-FTIR spectra of fresh and aged SiO_2/PEI samples (PEI loading 1 $g_{PEI}\ g_{SiO2}^{-1}$).

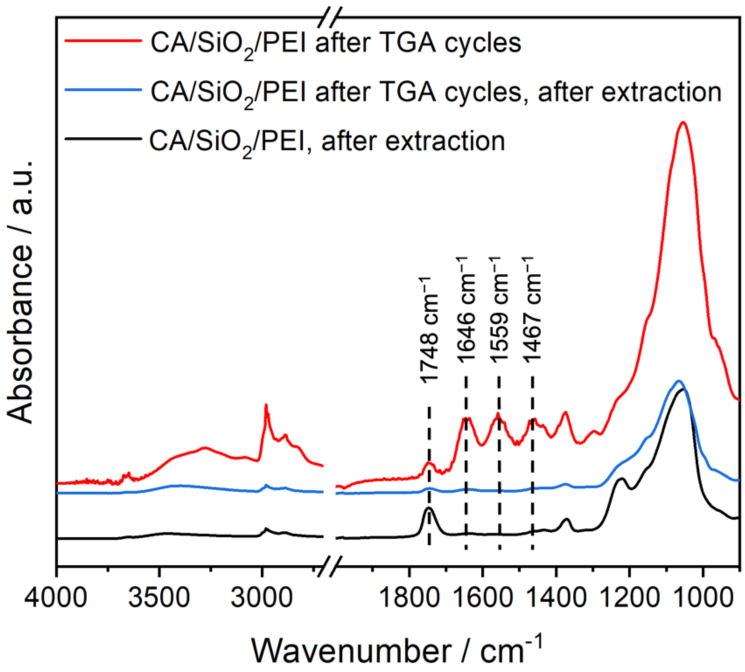

Figure S10. ATR-FTIR spectra of CA/SiO_2/PEI (1.0 g_{PEI} g_{SiO2}^{-1}) after MeOH extraction, CA/SiO_2/PEI after CO_2 sorption cycles, and CA/SiO_2/PEI after CO_2 sorption cycles plus MeOH extraction.

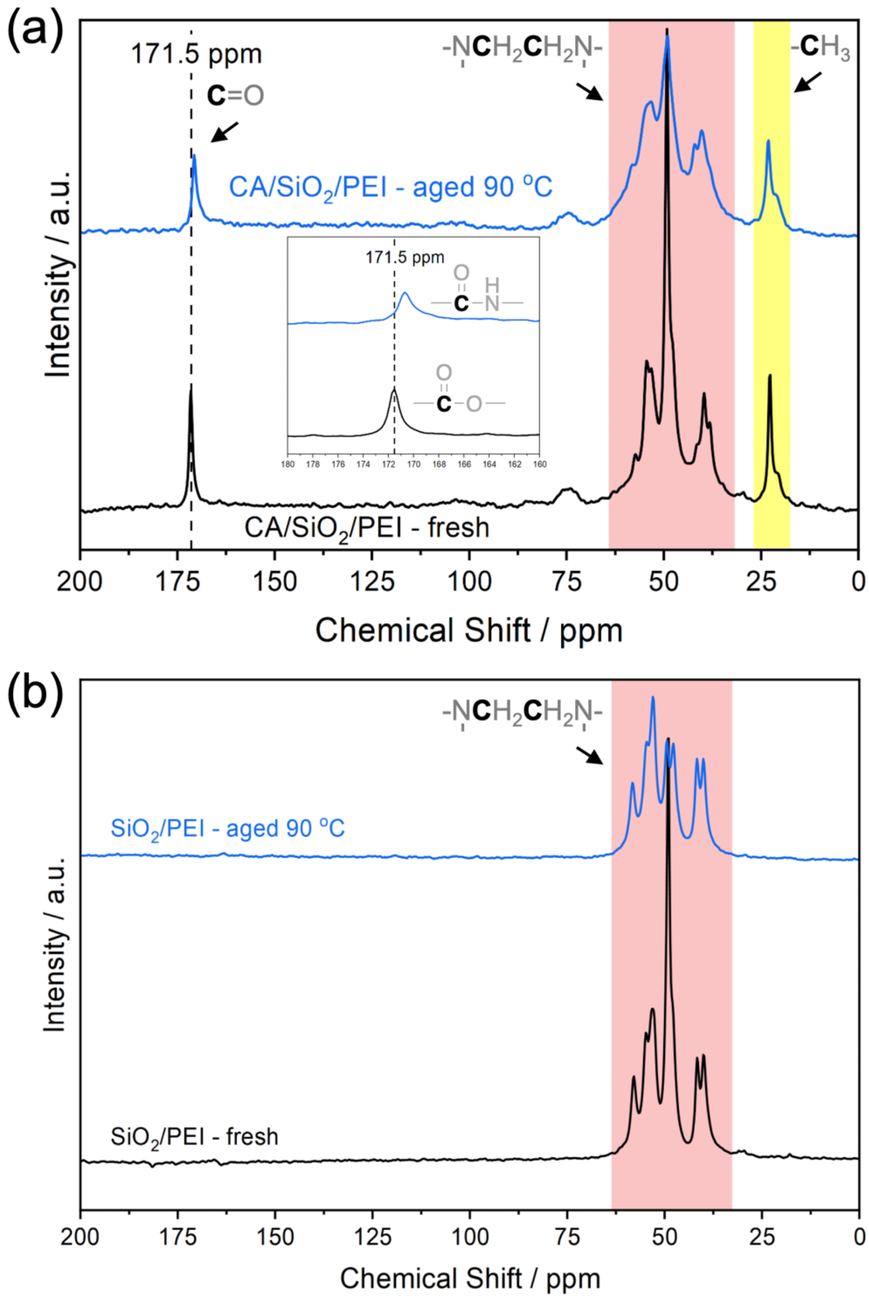

Figure S11. Solid State ^{13}C-NMR spectra of freshly prepared and thermally cycled (a) CA/SiO_2/PEI (1.0 g_{PEI} g_{SiO2}^{-1}) and (b) SiO_2/PEI (1.0 g_{PEI} g_{SiO2}^{-1}) samples. The inset of Figure S11a highlights the peak shift of C=O carbon after aging.

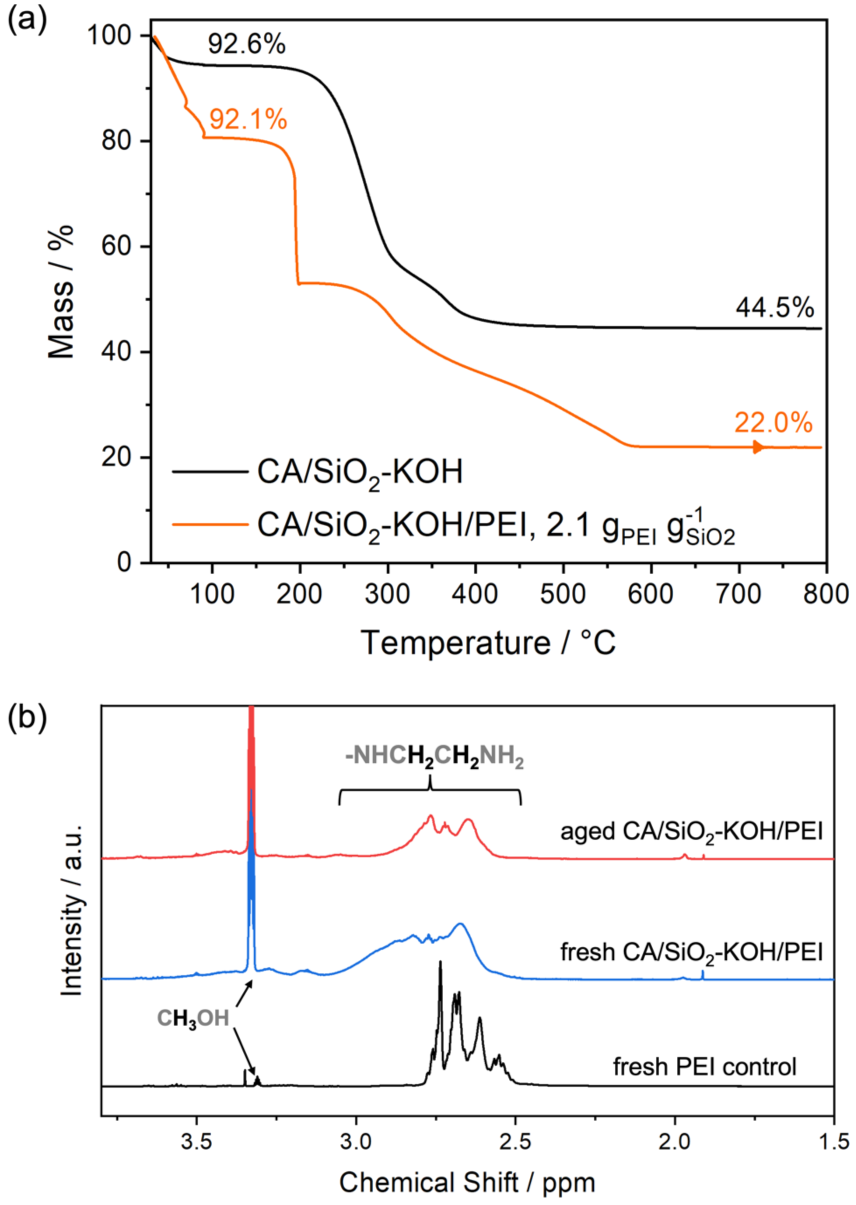

Figure S12. (a) The TGA trace of CA/SiO_2-KOH before and after PEI loading. (b) ^{1}H-NMR spectra of MeOH-d_4 extraction of CA/SiO_2-KOH/PEI. The ^{1}H-NMR spectrum of fresh PEI dissolved in MeOH-d4 is shown for comparison.

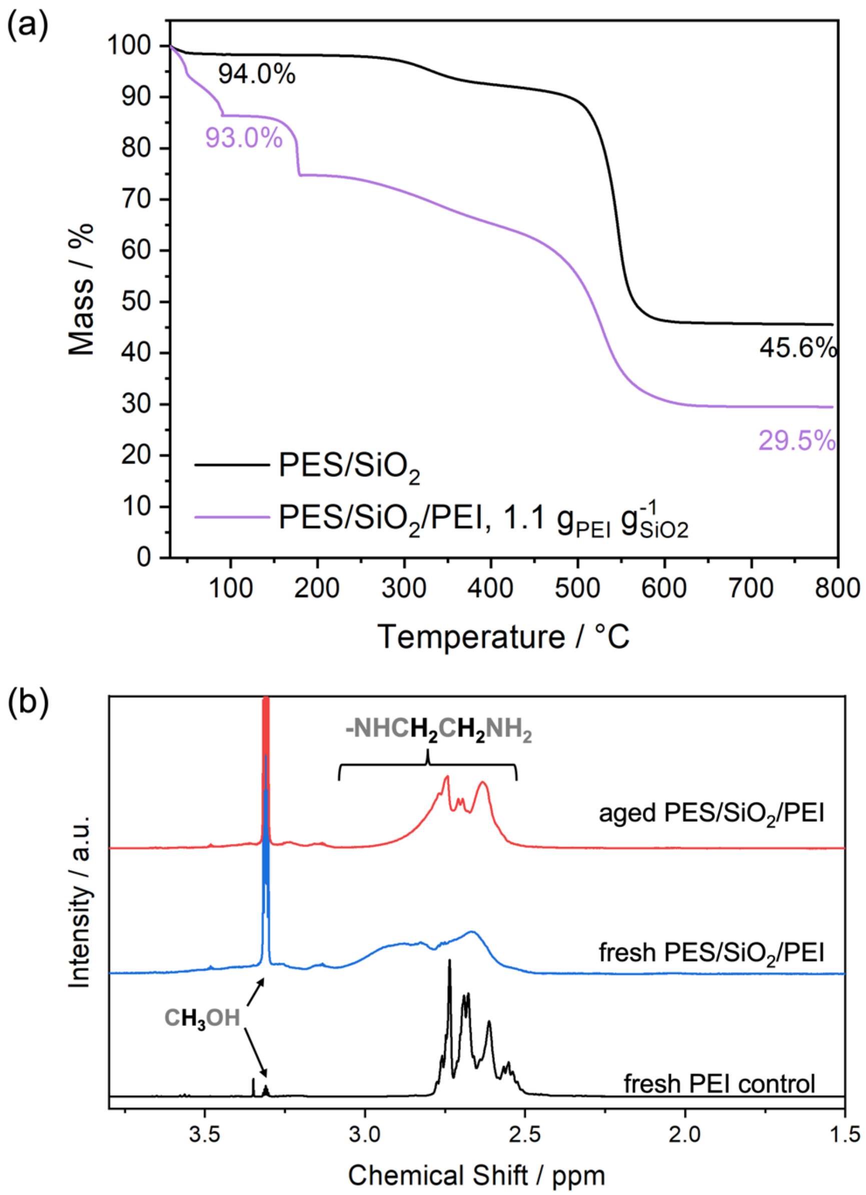

Figure S13. (a) The TGA trace of PES/SiO_2 before and after PEI loading. (b) ^{1}H-NMR spectra of MeOH-d_4 extraction of $PES/SiO_2/PEI$. The ^{1}H-NMR spectrum of fresh PEI dissolved in MeOH-d4 is shown for comparison.

Table S2. CO_2 working capacities of CA/SiO_2/PEI in the CO_2 cyclic sorption experiments.

CO_2 working capacity / mmol g^{-1}	CA/SiO_2/PEI, 1 g_{PEI} g_{SiO2}^{-1}	CA/SiO_2/PEI, 0.46 g_{PEI} g_{SiO2}^{-1}	CA/SiO_2/PEI, 1 g_{PEI} g_{SiO2}^{-1}, after 110 °C activation in vacuum
Cycle 1	1.06	1.15	0.55
Cycle 2	1.02	0.95	0.54
Cycle 3	0.98	0.88	0.53
Cycle 4	0.92	0.83	0.52
Cycle 5	0.9	0.77	0.51
Cycle 6	0.86	0.74	0.50
Cycle 7	0.83	0.69	0.50
Cycle 8	0.81	0.65	0.49
Cycle 9	0.79	0.62	0.48
Cycle 10	0.77	0.60	0.47
Cycle 11	0.75	0.58	0.47
Cycle 12	0.73	0.56	0.47
Cycle 13	0.72	0.54	0.46
Cycle 14	0.71	0.52	0.46
Cycle 15	0.7	0.51	0.46
Cycle 16	0.69	0.49	0.46
Cycle 17	0.68	0.48	0.46
Cycle 18	0.65	0.47	0.45
Cycle 19	0.65	0.46	0.44
Cycle 20	0.64	0.45	0.44

Table S3. CO_2 working capacities of CA/SiO_2-KOH/PEI and PES/SiO_2/PEI in the CO_2 cyclic sorption experiments.

CO_2 working capacity / mmol g^{-1}	CA/SiO_2-KOH/PEI	PES/SiO_2/PEI*
Cycle 1	1.64	1.33
Cycle 2	1.77	1.50
Cycle 3	1.76	1.48
Cycle 4	1.73	1.48
Cycle 5	1.7	1.47
Cycle 6	1.69	1.48
Cycle 7	1.67	1.47
Cycle 8	1.66	1.47
Cycle 9	1.65	1.47
Cycle 10	1.63	1.47
Cycle 11	1.62	1.46
Cycle 12	1.61	1.46
Cycle 13	1.6	1.46
Cycle 14	1.59	1.47
Cycle 15	1.58	1.46
Cycle 16	1.57	1.46
Cycle 17	1.56	1.46
Cycle 18	1.55	1.46
Cycle 19	1.54	1.46
Cycle 20	1.51	1.46
Cycle 21	-	1.46
Cycle 22	-	1.46

* The CO_2 working capacities of PES/SiO_2/PEI were obtained after aging in 400 ppm CO_2 and 2.0% H_2O at 90 °C for 300 min except for the first two cycles.

9. References

(1) Sujan, A. R.; Pang, S. H.; Zhu, G.; Jones, C. W.; Lively, R. P. Direct CO_2 Capture from Air using Poly(ethylenimine)-Loaded Polymer/Silica Fiber Sorbents. *ACS Sustainable Chem. Eng.* **2019**, *7*, 5264-5273. 10.1021/acssuschemeng.8b06203

(2) Labreche, Y.; Lively, R. P.; Rezaei, F.; Chen, G.; Jones, C. W.; Koros, W. J. Post-spinning infusion of poly(ethyleneimine) into polymer/silica hollow fiber sorbents for carbon dioxide capture. *Chem. Eng. J.* **2013**, *221*, 166-175. 10.1016/j.cej.2013.01.086

(3) Labreche, Y.; Fan, Y.; Rezaei, F.; Lively, R. P.; Jones, C. W.; Koros, W. J. Poly(amide-imide)/Silica Supported PEI Hollow Fiber Sorbents for Postcombustion CO_2 Capture by RTSA. *ACS Appl. Mater. Interfaces* **2014**, *6*, 19336-19346. 10.1021/am505419w

(4) Rim, G.; Priyadarshini, P.; Song, M.; Wang, Y.; Bai, A.; Realff, M. J.; Lively, R. P.; Jones, C. W. Support Pore Structure and Composition Strongly Influence the Direct Air Capture of CO_2 on Supported Amines. *J. Am. Chem. Soc.* **2023**, *145*, 7190-7204. 10.1021/jacs.2c12707

(5) Holmes, H. E.; Ghosh, S.; Li, C.; Kalyanaraman, J.; Realff, M. J.; Weston, S. C.; Lively, R. P. Optimum relative humidity enhances CO_2 uptake in diamine-appended M_2(dobpdc). *Chem. Eng. J.* **2023**, *477*, 147119. 10.1016/j.cej.2023.147119
